# Isolation of a potential probiotic strain *Bacillus amyloliquefaciens* LPB‐18 and identification of antimicrobial compounds responsible for inhibition of food‐borne pathogens

**DOI:** 10.1002/fsn3.3094

**Published:** 2022-12-12

**Authors:** Hedong Lu, Panping Yang, Mengyuan Zhong, Muhammad Bilal, Hai Xu, Qihan Zhang, Jiangnan Xu, Naiguo Liang, Shuai Liu, Li Zhao, Yuping Zhao, Chengxin Geng

**Affiliations:** ^1^ School of Life Science and Food Engineering Huaiyin Institute of Technology Huaian China; ^2^ National Engineering Research Center for Functional Food Jiangnan University Wuxi China

**Keywords:** antagonism, *Bacillus amyloliquefaciens*, fengycin, lipopeptides, probiotics

## Abstract

This study was carried out to screen a potential probiotic microbe with broad‐spectrum antagonistic activity against food‐borne pathogens and identify the antimicrobial compounds. Based on morphological and molecular analysis, a new *Bacillus* strain with the ability to produce effective antimicrobial agents was isolated from the breeding soil of earthworms and identified as having a close evolutionary footprint to *Bacillus amyloliquefaciens*. The antimicrobial substances produced by *B. amyloliquefaciens* show effective inhibition of *Aspergillus flavus* and *Fusarium oxysporum* in an agar diffusion assay. Antimicrobial agents were identified as a series of fengycin and its isoforms (fengycin A and fengycin B) after being submitted to RT‐HPLC and MALDI‐TOF MS analyses. To evaluate the probiotic activity of the *B. amyloliquefaciens*, antibiotic safety and viability of the isolated strain in a simulated gastrointestinal environment were carried out. The safety test result revealed that strain LPB‐18 is susceptible to multiple common antibiotics. Moreover, acidic condition and bile salts assay were carried out, and the results revealed that it couble be a potential probiotic microbe *B. amyloliquefaciens* LPB‐18 is good choice for biological strains in agricultural commodities and animal feedstuffs.

## Graphical abstract

We isolated potential probiotic microbe with broad‐spectrum antagonistic activity against food‐borne pathogens and identified the antimicrobial compounds which belong to fengycins by RT‐HPLC and MALDI‐TOF MS analyses. Meanwhile, we also evaluated the probiotic activity of the strain.

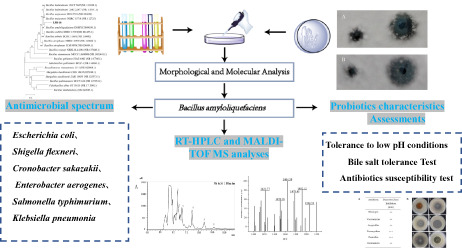

## 1 INTRODUCTION

Mycotoxins are toxic secondary metabolites produced by filamentous fungi (molds), most often *Aspergillus* and *Fusarium* (Nesic et al., 2021). Mycotoxins can contaminate preharvest and postharvest crops and pose significant safety threats to human foods and animal feedstuffs in direct contaminating agricultural commodities or through a “carry‐out” mechanism after feeding toxic feedstuffs to an animal (Emmanuel et al., 2020). It has been published that 25% of world grain crop was contaminated with mycotoxins by the Rapid Alert System for Food and Feed of EU (RASFF) (Agriopoulou et al., 2020; Milićević et al., 2016). Among all mycotoxins, aflatoxins (AFs) are a group of structurally similar, poisonous secondary metabolites produced mainly by *A. flavus*, which may grow and colonize on a variety of foods, including cereals, peanuts, almonds, cottonseeds, groundnuts, and milk and can be affected by aflatoxins M1 (AFM1) (Eskola et al., 2020; Pitt & Miller, 2017). The AFs could induce various adverse health effects, including immunosuppressive effects, mutagenic and carcinogenic effects on the liver. Zearalenone (ZEN), an estrogenic mycotoxin, is produced by *Fusarium* species, mainly *F. graminearum and F. oxysporum*. ZEN is frequently detected on wheat, corn, and sorghum. It has been published that ZEN significantly distorts the female reproductive system by competitive binding to estrogen receptors (Bryła et al., 2018). Furthermore, *A. flavus* and *F. oxysporum* have caused nonnegligible food safety and public health troubles.

During the long‐term struggle with mycotoxins contamination, biological control agents are demonstrated as promising candidates for food safety and efficiency. The strategy is conducted by applying microbial antagonists or competitors that can completely limit the growth of toxigenic molds. Recently, some reports showed that *B. amyloliquefaciens* can strongly suppress the growth of *Fusarium* and *Aspergillus*. For example, *B. amyloliquefaciens* PPL exhibited significant antifungal activity of *Fusarium and Colletotrichum*, which cause defoliation and loss yield of crops by pathogens penetrating plant tissues (Kang et al., 2020). *B. amyloliquefaciens* SYBC H47 isolated from raw honey, and demonstrated significant antifungal activity against *Aspergillus niger* (Li et al., 2016)*. B. amyloliquefaciens* LYZ69 from healthy alfalfa roots demonstrated strong antifungal activity against *Colletotrichum truncatum* by producing bacillomycin D and fengycin, which caused oxygen radical accumulation and induced apoptosis‐like cell death in *C. truncatum hyphae* (Hu et al., 2021). *B. amyloliquefaciens* FZB42 is well known for its potential as a biocontrol strain against *F. graminearum* (Hanif et al., 2019). Lee et al. (2017) isolated a *B. amyloliquefaciens* from moldy corn samples which exhibited high ZEN removal capability. Furthermore, *B. amyloliquefaciens* had been generally classified as GRAS (generally regarded as safe) by the U.S. Food and Drug Administration (FDA), which has broad applicant in beverage and dairy products fermentation, synthesis and/or hydrolysis of food compounds as a promising potential probiotic (Berikashvili et al., 2018; Chen et al., 2020; WoldemariamYohannes et al., 2020).

In this study, we isolated a *B. amyloliquefaciens* strain with significant inhibition against *A. flavus* and *F. oxysporum* and identified antimicrobial components by Reversed‐phase high‐performance liquid chromatography (RT‐HPLC) and MALDI‐TOF MS analysis, respectively. Moreover, we also evaluated the probiotic characteristics of the *B. amyloliquefaciens* by bile salt tolerance test and tolerance to low pH conditions, and antibiotic safety.

## 2 MATERIALS AND METHODS

### 2.1 Microbial strains, chemicals, and reagents

The soil samples in this experiment were collected from the breeding soil of earthworms. In this study, the indicator strains were preserved in the laboratory. Acetonitrile, trifluoroacetic acid, and methanol were highly pure and were purchased from Tedia. Other related biochemical reagents were obtained from Sinopharm Chemical Reagent Co., Ltd. A high‐performance liquid phase preparation system of Waters was purchased from Waters Inc.

### 2.2 Soil sample collection from breeding soil of earthworm

To isolate antagonistic strains against *A. flavus* and *F. oxysporum*, the soil samples were collected in Huaian County, Jiangsu Province, China. The humidity and temperature of the collection site were 80% RH and 29°C, and the site is located at 119°0′72″E, 33°4′73″S. The nonsterile soil sample was collected from breeding soil of earthworm habitation and kept in cold storage at 4°C.

### 2.3 Isolation of *Bacillus* strains

The soil sample disposing was performed as previously described (Xiao et al., 2021). Briefly, the sample remained 30 min in a boiling water bath (60°C), and an aliquot of sludge sample (0.5 g) was suspended with 50‐ml sterile water in a 250‐ml conical flask shaken at 180 rpm for 1 h at 33°C. Serial dilutions from 10^−1^ to 10^−6^ were prepared using sterile water. The diluents of 10^−4^, 10^−5^, and 10^−6^ were plated on Luria‐Bertani (LB, Shanghai, China) medium (10 g/L, peptone; 5 g/L, yeast extract, and NaCl, respectively, pH 7.0) and incubated aerobically at 33°C on constant incubator for 24 h. A single colony was preserved and numbered. The dual culture method was used to screen the strain with antifungal activity to detect the in vitro antagonistic activity of the strain against *A. flavus* and *F. oxysporum*, and incubated at 28°C for 7 days. If the isolated strain can produce substances with antifungal activity against *A. flavus* and *F. oxysporum*, an inhibition zone will arise around the indicator of the colony of the strain.

### 2.4 Measurement strains of antibiotic resistance and antagonistic activity against pathogens

Antibiotic resistance and antagonistic activity against pathogens were determined according to the agar disk diffusion assay (Selvin et al., 2020). Briefly, Sterile Oxford cups (6 mm × 8 mm × 10 mm, inner diameter × outer diameter × height) were placed on the assay medium seeded with the fresh culture suspension of different indicator strains (about 10^8^ colony‐forming units/ml for bacterial cells and 10^6^ spores/ml for fungal strains). The same amount of sterile water was used as the negative control. Besides, the same amount of sterile water, anhydrous ethanol, DMSO, and 0.01 mol/L HCl applied to prepare antibiotics were used as a negative control to subtract the inhibitory activity of the solvents. After incubation for 24 h at 33°C for bacteria and 7 days at 28°C for fungal strains, the inhibition zones were measured and recorded as a mean diameter (mm). All tests were conducted in triplicate. Antagonistic activity against pathogenic fungi and bacteria was determined according to the agar disk diffusion assay.

### 2.5 Identification of Bacillus strains

#### 2.5.1 Biochemical tests

The morphological properties were examined by electron microscopic. Biochemical and physiological properties of the goal strains were carried out according to the bacterial identification program MS(i)/C005‐C01 (Bergey's Manual of Determinative Bacteriology [9th Edition], R.E. Buchanan et al., Science Press 2).

#### 2.5.2 Molecular identification

16S rDNA and gene polymerase chain reaction (PCR) and sequencing Genomic DNA from the strain was extracted using Omega ENZA Bacterial DNA Kit (D3350‐02 Omega Bio‐Norcross) according to the manufacturer's instruction (Efe, 2020). The 16S rRNA gene was amplified using universal primers (Table 1). The amplified product was purified with the SanPrep Column PCR Product Purification Kit (B518131‐0050) (Sangon Biotech) and sent for sequencing to GenScript. The sequences obtained were compared with the previously sequenced gene in the GenBank database using the National Center for Biotechnology Information's Blast search program (Bethesda, United States). The most closely related sequences of strain types were aligned using Clustal software, and phylogenetic trees were constructed in MEGA version 11 using the Neighbor‐joining method.

**TABLE 1:** Primers used in the experiment

Genes	Primers
16S rDNA (KM117160.1)	F: 5′‐AGAGTTTGATCCTGGCTCA‐3′
	R: 5′‐GGTTACCTTGTTACGACTT‐3′
*fenC* (AF087452.1)	F: 5′‐TTTGAAAGAAAATACTTAGGTTAA‐3′
	R: 5′‐AACTTTCTTTTATGAATACCAAATT‐3′
*ituA* (D21876.1)	F: 5′‐ATGAAAATTTACGGAGTATATATG‐3′
	R: 5′‐TTATAACAGCTCTTCATACGTT‐3′
*srf* (EU882341.1)	R: 5′‐ATGAAGATTTACGGAATTTATATG‐3′
	F: 5′‐TTATAAAAGCTCTTCGTACGAG‐3′
*bmy D* (12963911)	F: 5′‐TTCAGGATGCCGTTACACTTG‐3′
	R: 5′‐GAAATCACATGGATGCCGTTCTTC‐3′

[corrections added on 13 January 2023, after the first online publication: values for bmy D were corrected]

### 2.6 Cloning of lipopeptide genes by PCR analysis

The genomic DNA was extracted through Omega ENZA Bacterial DNA Kit according to the manual. Specific primers synthase gene of lipopeptides: *fenC* (fengycin), *ituA* (iturin A), *srf* (surfactin), and *bmy D* (bacillomycin D) are listed in Table 1. PCR amplification performed with the same system as the 16S rDNA sequence amplification system: denaturation at 94°C for 3 min, final denaturation at 94°C for 50 s, primer annealing at 55°C for 50 s, and an ultimate extension of 10 min at 72°C. The PCR products were subjected to gel‐purification and ligated to pUcm‐T vector, as described previously (Zhao et al., 2013). After electro‐transformation into *E. coli* DH5α with competent cells, the positive recombinants were chosen in LB agar plates supplemented with kanamycin. After further identification by colonial PCR, the aimed plasmid was extracted and submitted to sequence by GenScript (Nanjing, China). The sequences obtained were compared with the previously sequenced genes in the GenBank database using the National Center for Biotechnology Information's Blast search program (Bethesda, United States; http://www.ncbi.nlm.nih.gov).

### 2.7 Isolation and purification of antimicrobial substances from *B. amyloliquefaciens* LPB‐18

To produce the antimicrobial substances, the strain LPB‐18 was inoculated into LB broth medium at 33°C and 180 r/min for 16 h (approximately 5 × 10^7^ CFU/ml) to prepare seed cultures. A 3% (v/v) of seed cultures was transferred into a 1‐L shake flask containing 500 ml of fermentation medium (20 g/L, yeast extract 1 g/L, l‐glutamic acid 5 g/L, KCl 0.5 g/L, MgSO_4_ 0.5 g/L, KH_2_PO_4_ 1 g/L, l‐phenylalanine 2 mg/L, MnSO_4_ 5 mg/L, FeSO_4_ 0.15 mg/L, CuSO_4_ 0.16 mg/L) at 33°C and 180 r/min for 72 h. After fermentation, the culture was centrifuged at 10,000 *g* for 15 min at 4°C to make the cell‐free supernatants (CFS), which were precipitated using 6 mol/L HCl to pH 2 and stored overnight at 4°C. The acid precipitate of cell‐free broth was then collected by centrifuging at 10,000 for 15 min at 4°C, discarding the supernatant, and the precipitate was dissolved with methanol, which was further neutralized to pH 7.0 using 1 N NaOH. The crude extract was stored at 4°C, designated as Anti LPB‐18. The agar disk diffusion assay experiments above were also implemented to verify whether the antimicrobial substances were sufficiently extracted.

### 2.8 RT‐HPLC analysis

The crude extract was filtered through a 0.22 μm pore filter, and 30‐μl aliquot was injected into an Eclipse XDB‐C18 column (5 μm, 250 × 4.6 mm, Agilent) in the RT‐HPLC system (Shimadzu, Japan) for further identification and purification. The mobile phase was deionized water containing 0.1% trifluoroacetic acid, and mobile phase B was acetonitrile containing 0.1% trifluoroacetic acid. The flow rate was maintained to be 6 ml/min under the following conditions: 0–15 min, 30% A to 45% A, 70% B to 55% B; 15–55 min, 45% A–55% A, 55% B–45% B. Monitoring was conducted at 205‐nm ultraviolet detector.

### 2.9 Mass spectrometry analysis of antimicrobial agents

To obtain molecular information on antimicrobial agents further, the extraction was subjected to the MALDI‐TOF MS instrument (Bruker Daltonics). After RT‐HPLC analysis, the agents corresponding to every peak pattern were separated and antagonistic activities were verified according to the agar disk diffusion assay. The *m*/*z* values of antimicrobial agents were measured from 150 to 2000 Da. All tested samples (2 μl) were mixed with an equal volume of the matrix (a saturated solution of α‐cyano‐4‐hydroxycinnamic acid in 50% acetonitrile with 0.1% TFA), as described previously.

### 2.10 Probiotics characteristics assessments of the strain LPB‐18

#### 2.10.1 Tolerance to low‐pH conditions

This assay was performed as previously described (Leite et al., 2015). Briefly, the seed medium of strain LPB‐18 was grown at 33°C for 12 h (about 10^6^ colony/ml for bacterial), and 2‐ml culture was harvested by centrifugation at 8000 rpm for 10 min. After the phosphate‐buffered saline solution (PBS) buffer was prepared, cells were washed thrice in sterile PBS and resuspended for obtaining an optical density of 0.5 at 600 nm. The suspensions were adjusted to pH 2.0, pH 3.0, pH 4.0 with HCI and incubated statically at 33°C for 3 h. During the incubation period, 2‐ml sample was tested at 0, 1, 2, and 3 h, respectively. The strain LPB‐18 tolerance to low pH conditions was estimated by using a blood counting chamber, and the percentage of cell survival rate was counted using the following equation:

(1) $$\text{Survival rate}\;\left(\%\right)=\frac{\text{Final}\;\left(\mathrm{Log}\frac{\mathrm{CFU}}{\mathrm{ml}}\right)}{\text{Initial}\;\left(\mathrm{Log}\frac{\mathrm{CFU}}{\mathrm{ml}}\right)}\times 100\%$$

#### 2.10.2 Bile salt tolerance test

This assay was carried out according to Talib et al. (2019), with slight modification. Briefly, the seed medium of strain LPB‐18 was grown at 33°C for 12 h (about 10^6^ colony/mL for bacterial), and 2‐ml culture was harvested by centrifugation at 8000 rpm for 10 min. The cell was washed thrice in sterile PBS and resuspended for obtaining an optical density of 0.5 at 600 nm. The suspensions were adjusted to 0.3% (w/v) ox gall and 0.5% ox gall (Sigma), and incubated statically at 33°C for 3 h. During the incubation period, 2‐ml sample was counted at 0, 1, 2, and 3 h, respectively, and the percentage of cell survival rate was counted using equation (1).

### 2.11 Statistical analysis

The diameters of inhibition zone of isolated strain against many pathogenic indicators were expressed as mean values ± SD. All the assays were repeated thrice at least, and the statistical was subjected to a *t*‐test significant difference analysis.

## 3 RESULTS

### 3.1 Isolation and screening of antagonistic bacteria of *Aspergillus flavus* and *Fusarium oxysporum*

**FIGURE 1:**
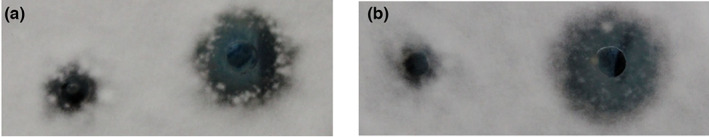
Inhibition zone of *Aspergillus flavus* (a); inhibition zone of *Fusarium oxysporum* (b)

The PDA plates containing the pathogens *A. flavus* and *F. oxysporum* were essential for screening antagonistic pathogens. To obtain the antagonistic strains against *A. flavus* and *F. oxysporum*, 20 soil samples were collected from the earthworm habitation (Huaian city, Jiangsu Province, China). After disposing of the soil sample in a boiling water bath (60°C), 83 different strains were isolated and purified, and their inhibitory ability was determined with screening plates. Among them, strain LPB‐18 showed potent antifungal activity with inhibitory circle diameters of 14.980 ± 0.125 mm and 15.623 ± 0.215 mm against *A. flavus* and *F. oxysporum*, respectively (Figure 1a,b). The strain LPB‐18 was the only one that simultaneously antagonized pathogenic fungi *A. flavus* and *F. oxysporum*. Therefore, it was selected for further investigation.

### 3.2 The antagonistic activity against pathogens of the strain LPB‐18

**FIGURE 2:**
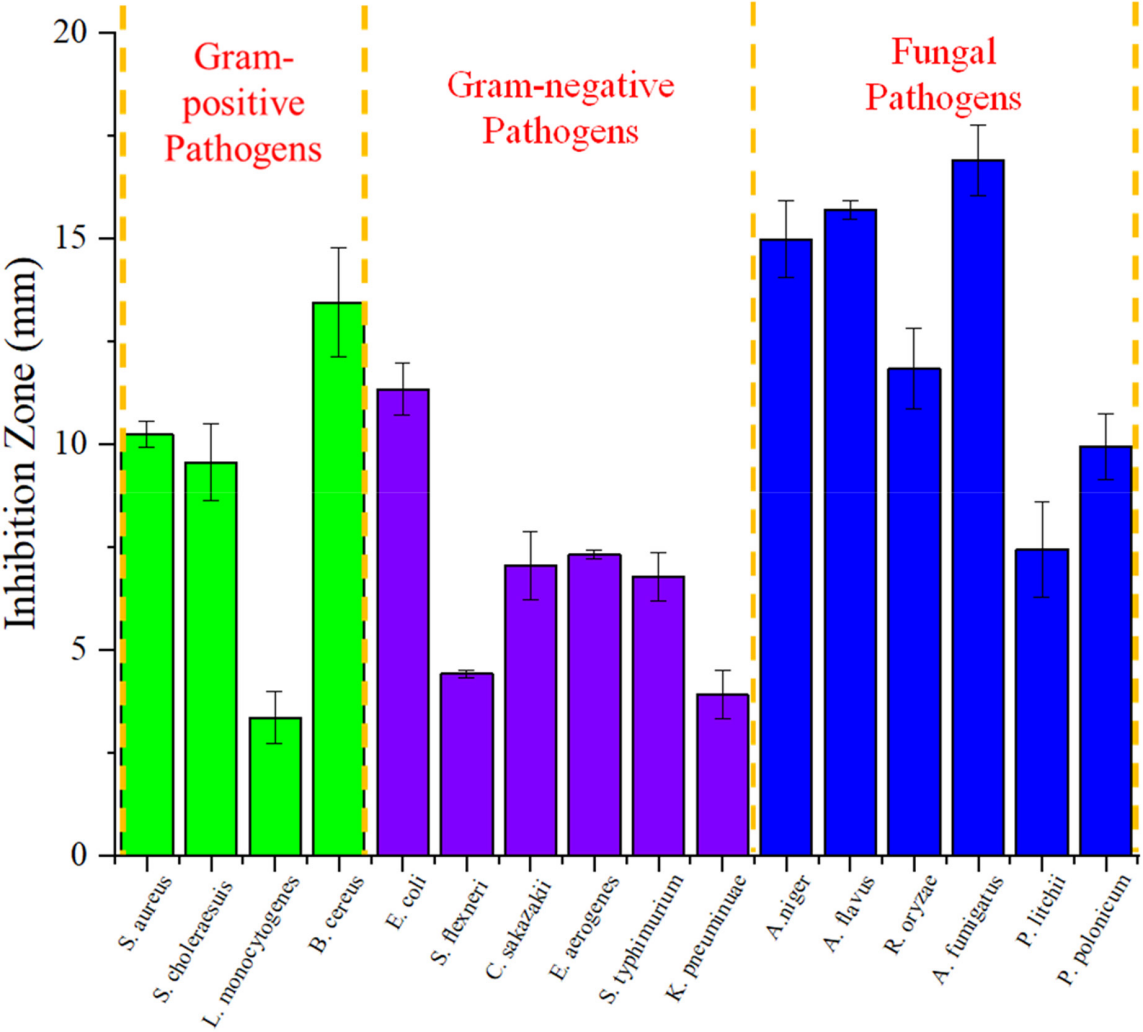
Antimicrobial spectrum of cell‐free supernatants from *B. amyloliquefaciens* LPB‐18.

To determine the antimicrobial spectrum of the strain LPB‐18 fermentation products, 16 kinds of food‐borne pathogens were used as indicators, and the agar disk diffusion assay was performed. As shown in Figure 2, the supernatant of strain LPB‐18 exhibited significant inhibitory against Gram‐positive and Gram‐positive pathogens and the most potent antagonistic effects on fungal pathogens, especially filamentous fungi. The inhibition circle diameters of 10.24, 9.56, 3.36, and 13.45 mm against *Staphylococcus aureus*, *Salmonella choleraesuis*, *Listeria monocytogenes*, and *Bacillus cereus*, respectively. The inhibition circle diameters of gram‐positive pathogens of 11.34, 4.42, 7.05, 7.73, 6.79, and 3.92 mm against *Escherichia coli*, *Shigella flexneri*, *Cronobacter sakazakii*, *Enterobacter aerogenes*, *Salmonella typhimurium*, and *Klebsiella pneumonia*, respectively. The supernatant of strain LPB‐18 showed the biggest inhibition zones of 14.98 and 15.62 mm against *A. flavus* and *F. oxysporum*, respectively. The strain LPB‐18 exhibited significant antimicrobial activity and have promising potential to be a candidate for alternative antibiotics.

### 3.3 Antibiotics susceptibility test

**FIGURE 3:**
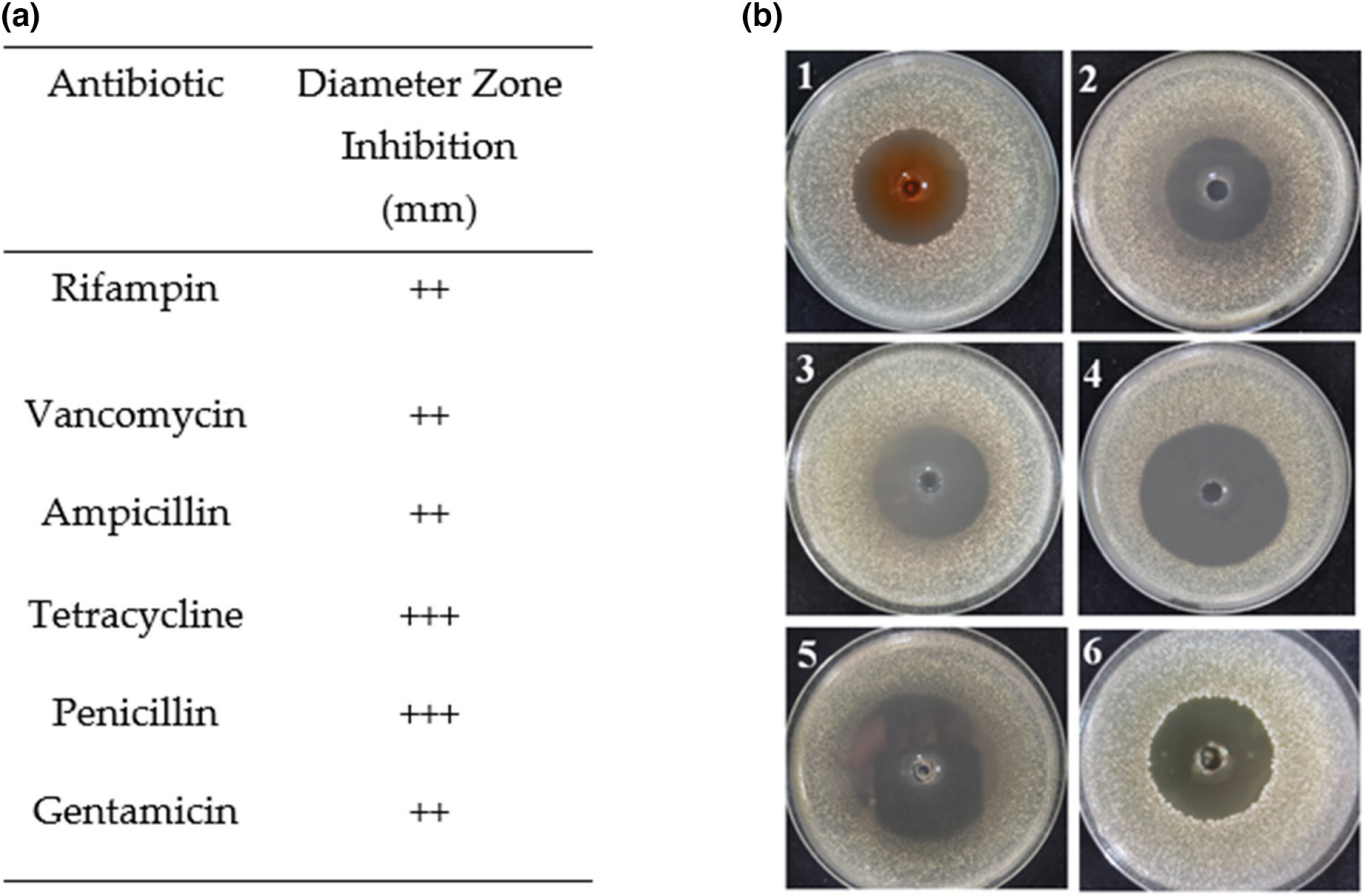
Antibiotic susceptibility test using disk diffusion on LB agar. Figure 3a Susceptibility test result of antibiotics, where rifampicin (1), gentamicin (2), tetracycline (3), penicillin (4), ampicillin (5), and vancomycin (6) antibiotics contain zones of inhabitation. Values indicate mean of triplicates. Resistant (−), moderately susceptible (+; inhibition zone: 10–20 mm), susceptible (++; inhibition zone: 21–30 mm), and very susceptible (+++; inhibition zone >31 mm).

Considering food safety and multidrug‐resistant pathogens, antibiotics susceptibility was analyzed by the agar disk diffusion assay by measuring the zone of inhibition toward gentamicin, vancomycin, ampicillin, rifampicin, tetracycline and penicillin, as shown in Figure 3b. The result of the assay was shown in Figure 3a and expressed as resistant (−), moderately susceptible (+), susceptible (++), and very susceptible (+++). The strain LPB‐18 was susceptible to gentamicin, rifampicin, vancomycin, ampicillin and very susceptible to tetracycline and penicillin with inhibition zones ranging between 33~55 mm.

### 3.4 Identification of bacterial strain and detection of genes related to lipopeptides

To obtain discriminated classification of the strain LPB‐18, genomic DNA was extracted using Omega ENZA Bacterial DNA Kit (Sangon Biotech, China) according to the manual, and biochemical and physiological properties were performed as described in the Methods section. The colonies of strain LPB‐18 inoculated on LB agar plates at 33°C for 24 h were white, smooth‐surface, central convex with irregular margins, and became dried and wrinkled with the prolongation of culture time. Cells of the strain LPB‐18 grow at 20°C–50°C (optimum temperature 33°C) in oxygen boosting in the presence of 1–9% (w/v) NaCl. Rather than Rhamnose, α‐lactose, and d‐xylose, other carbon source strains can be utilized (Table 2).

**TABLE 2:** Biochemical and physiological characteristics of strain LPB‐18

Characteristic	Results	Characteristic	Results
Gram's reaction	+	Maltose	+
Endospore formation	+	Saccharose	+
Indole test	−	Rhamnose	−
V‐P determination	+	Glucose	+
Litmus milk test	+	Fructose	+
Urease	−	Seminose	+
Starch hydrolysis	+	α‐lactose	−
NaCl tolerate	<10%	d‐xylose	−
Ribose	+	d‐sorbitol	+
Arabinose	+	Synanthrin	+
Sucrose	+	Growth between 20 and 50°C	+

**FIGURE 4:**
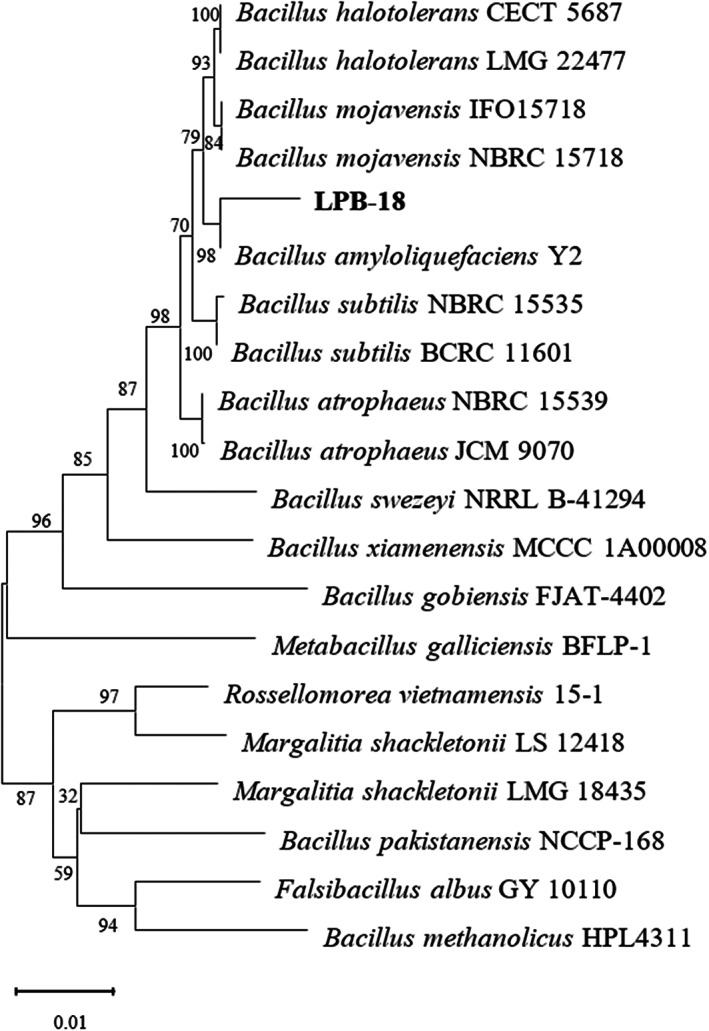
Neighbor‐joining phylogenetic tree based on 16S rDNA gene sequences

A 1450 bp region of the 16S rDNA sequence of strain LPB‐18 was determined and deposited with the sequences of NCBI databases, which showed high homology with *B. amyloliquefaciens* Y2 (99.45%). The phylogenetic tree based on the 16S rDNA sequences of strain LPB‐18 was designed and is shown in Figure 4. Four pairs of primers of lipopeptides (*fenC*, *srf*, *ituA*, and *bmy D*) were designed by primers 5 and synthesized for entrusting GenScript (Nanjing, China). Only *fenC* was detected by PCR analysis from *B. amyloliquefaciens* LPB‐18 (Table 3).

**TABLE 3:** PCR analysis of synthase gene antimicrobial lipopeptide

Term	*fenC*	*ituA*	*Srf*	*bmy D*
Result	+	−	−	−

### 3.5 Isolation and purification of antimicrobial substances produced by *B. amyloliquefaciens* LPB‐18

**FIGURE 5:**
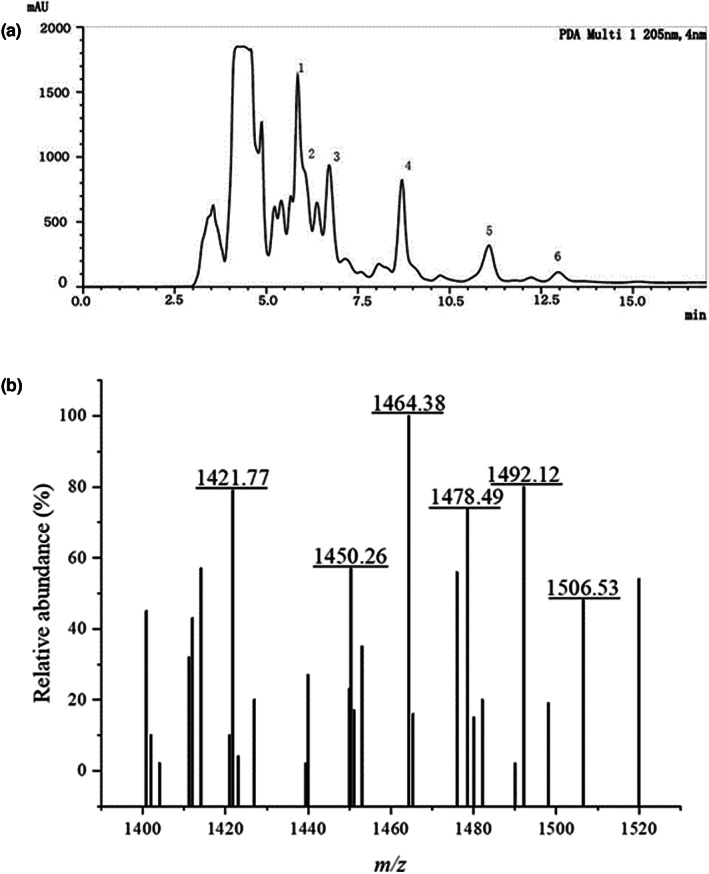
RT‐HPLC chromatogram analysis of lipopeptides mixture (a); MALDI‐ TOF MS analysis of lipopeptides mixtures (b).

To obtain and identify compositions with antibacterial activity from strain LPB‐18, the fermentation combination was separated and purified by acid precipitation, methanol extraction, and RT‐HPLC analysis (Figure 5a). The substances were examined by RT‐HPLC analysis at 205 nm after subjecting to agar diffusion assay. The agar disk diffusion assay of extraction from every peak revealed that the fractions with an elution time of 6–13 min contain antimicrobial agents.

### 3.6 Determination of antimicrobial agents with MALDI‐TOF MS

The MALDI‐TOF MS analysis was carried out to determine the molecular mass of antibacterial material. As shown in Figure 5b, the intense signals at *m*/*z* values range from 1450.26 to 1506.53 Da, which could be attributed to the isomers of fengycin, as previously described (Sarwar et al., 2018; Torres et al., 2015; Yang et al., 2015). In detail, the protonated molecular ion ([M + H] +) showed a set of ions of *m*/*z*:1450.26, 1464.38, 1478.49, 1492.12, and 1506.53 Da. A correspondent report had shown that the fengycin standard spectra peak at 1449.7, 1463.7, and 1477.7 Da were attributed to fengycin A isoforms, and spectra peak at 1491.7 and 1505.7 represented fengycin B isoforms. A tiny error was subjected to MALDI‐TOF MS, and the main peaks from the MS data were consistent with previous studies. The dominant antibacterial substance of strain LPB‐18 was identified as fengycin A (C_16_–C_17_) and fengycin B (C_15_–C_17_).

### 3.7 Probiotics characteristics assessments of the strain LPB‐18

**FIGURE 6:**
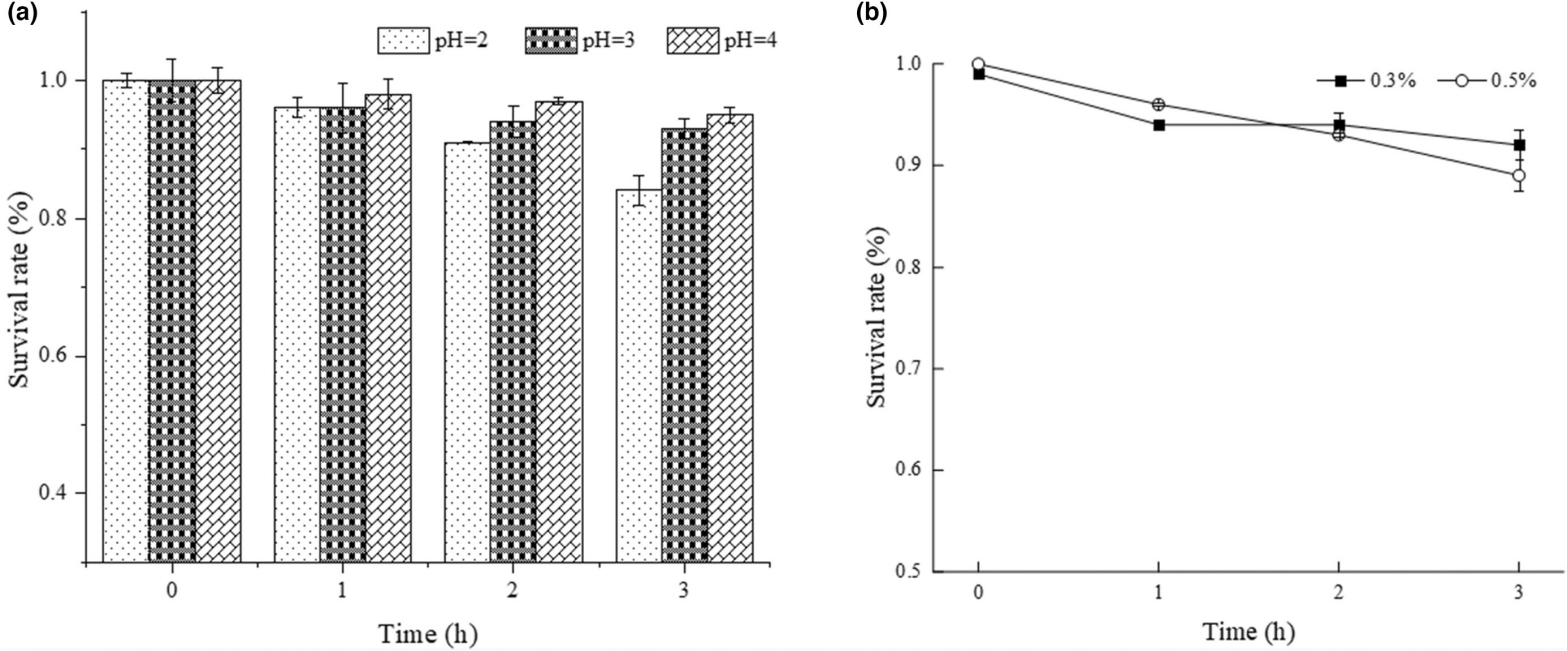
Survival rate of *B. amyloliquefaciens* LPB‐18 after incubation at acidic conditions (pH 2.0, pH 3.0 and pH 4.0) (a) and in the presence of 0.3% ((w/v) and 0.5% (w/v) bile salt (b).

The strain LPB‐18 probiotic attribute was evaluated by acidic and salt tolerance. As shown in Figure 6, *B. amyloliquefaciens* LPB‐18 was subjected to different acidic conditions (pH 2.0, pH 3.0, and pH 4.0) and presented a remarkable surviving rate of 84.32 ± 0.34% and 93.84 ± 0.61% after exposing to pH 2.0 and pH 3.0. As shown in Figure 5, the strain survival rate was counted with different levels (0.3 and 0.5) of bile salt disposing for 3 h, and it exhibited a great surviving rate of 92.65 ± 0.27% and 89.65 ± 0.75%, respectively. The results indicated that *B. amyloliquefaciens* LPB‐18 has great tolerance to acidic and salt conditions.

## 4 DISCUSSION

There are several approaches available for decontamination of mycotoxins in human foods and animal feeds, and these refer to chemical methods such as managing crops or cereals with fungicides or antibiotics or exposing containing food to acids, bases, and hydrogen peroxide; biological methods such as using natural antimicrobial substances to inhibit the growth of pathogenic and using adsorption or biotransformation agents to reduce and degrade mycotoxins to nontoxic forms (Ulger et al., 2020). Compared with chemical methods, biological methods are promising and rewarding due to a variety of advantages, such as efficiency, high specificity, nontoxicity, and the absence of harmful chemicals (Kabak et al., 2006). We isolated a *B. amyloliquefaciens* LPB‐18 with broad‐spectrum antimicrobial activity from the breeding soil of earthworms. Figure 2 shows that the supernatant of strain LPB‐18 fermentation broth had substantial inhibitory effects, particularly against filamentous fungi, for instance, the inhibition circle diameters of 14.98 and 15.69 mm against *A. flavus* and *F. oxysporum*, respectively. These findings suggested that *B. amyloliquefaciens* LPB‐18 has the ability to inhibit generation of mycotoxins.

In addition, the main antibacterial lipopeptide fengycins were identified by RT‐HPLC and MALDI‐TOF MS analysis and verified by synthase gene *fenC* of its synthase gena. It includes six elution peaks from 6 min to 13 min in RT‐HPLC analysis and six intense signals at *m*/*z* values range of 1450.26–1506.53 Da of MALDI‐TOF MS. According to previous reports, the lipopeptide was recognized as fengycin isoforms, including fengycin A (C_16_ **–**C_17_) and fengycin B (C_15_ **–**C_17_). Many studies have shown that fengycin has effective antifungal activity, especially filamentous pathogenic fungi (Gimenez et al., 2021; Lu et al., 2021), which was consistent with the strain LPB‐18 antimicrobial spectrum. The fengycin is composed of a β‐hydroxy fatty acyl chain and a decapeptide with eight kinds of amino acids and displays potent activity against filamentous fungi and bacteria (Tang et al., 2014; Vanittanakom et al., 1986). Coupled with this biological attributions is the promising prospect that has a great potential to be biorational fungicides (Lin et al., 2020; Zhang & Sun, 2018). Fengycin has many homologues. According to the length of the fatty acid chain, fengycin contains C_14_ ~ C_18_ carbon atoms. When classified from the amino acid composition of the cyclic peptide, fengycin was identified as fengycin A when the sixth amino acid was alanine and as fengycin B when the sixth amino acid was valine (Lin et al., 2020). Reports have demonstrated that fengycin can destroy the integrity of the cell wall and change the permeability of the cell membrane, which causes the cell's contents to leak out and lead to cell death (Lin et al., 2020; Piewngam et al., 2018). Besides that, fengycin can induce cell chromosome aggregation, reactive oxygen species (ROS) accumulation, mitochondrial membrane potential depolarization, phosphatidylserine externalization, DNA fragmentation, and cell apoptosis (Xiao et al., 2021). Fengycin, derived from *B. amyloliquefaciens* FZB42, suppresses *F. graminearum* growth by triggering its hyphae abnormalities and hyphal walls ruptured (Hanif et al., 2019).

The *Bacillus* species share the sporulation ability, which is a crucial feature for *Bacillus sp*. to survive harsh conditions of growth. Spore formers show vast tolerance and survivability in extreme temperatures, pH salt, and other hard state. The microbiota of the animal environment of intestines should be acid and bile salt resistant (Jezewska‐Frackowiak et al., 2018). To evaluate the strain probiotic potential, the bile salt tolerance test and tolerance to low pH conditions of *B. amyloliquefaciens* LPB‐18 were determined. It is an important precondition for potential probiotic strain resisting acidic conditions in the stomach and the bile salts in the duodenum (Fontana et al., 2013; Lee et al., 2017). In this study, *B. amyloliquefaciens* LPB‐18 survived 84% and 93% after exposure at pH 2.0 and pH 3.0 for 3 h. And the strain survival rate was counted with different levels (0.3 and 0.5) of bile salt disposing for 3 h, and it exhibited a great surviving rate of 92% and 89%. These results indicated that *B. amyloliquefaciens* LPB‐18 might be transited through the stomach, survive in the intestinal tract, and work effectively. Despite the beneficial features of *Bacillus* strains belonging to the safety group, a number of strains could pose a substantial health risk, carrying genes for various toxins or antibiotic resistance (Gut et al., 2018). In the past few years, antibiotics have served as an unprecedented role in saving millions of lives of bacterial infection (Brown & Wright, 2016; European Food Safety et al., 2018). However, the antibiotic abuse pushing the emergence of antibiotic‐refractory pathogens and the traditional therapeutic strategy severely nullifying the effects of life‐saving drugs (Liu et al., 2021). The World Health Organization (WHO) reported that multidrug‐resistant microbial pathogens have caused approximately 700,000 annal deaths (Samreen et al., 2021). Therefore, the AMR has become a serious global and it is an urgent need for alternatives to antibiotics. It is essential to determine antibiotic tolerance of *B. amyloliquefaciens* LPB‐18, which is a prerequisite for its use in animal feed. And the result exhibited that it is susceptible for antibiotics.

Recently, some other species strains were reported to have mycotoxins degrading ability. Bacon et al. reported that the endophytic *B. subtilis* has the ability to reduce mycotoxins accumulation (Bacon et al., 2001). Farzaneh et al. obtained a promising *A. flavus R5* antagonist strain *B. subtilis* UTBSP1, which can reduce the growth of *A. flavus R5* and aflatoxins accumulation (Farzaneh et al., 2016). Additives et al. displayed the strain *Eubacterium* BBSH 797, isolated from bovine rumen fluid, can transform the deoxynivalenol (DON) to nontoxic metabolite trichothecenes due to its epoxidase (Additives et al., 2017). Li et al. found that *B. velezensis* from the surface of a healthy race significantly controls *A. flavus* contamination (Li et al. 2022). Despite the many publications shown biocontrol or biodegradation of mycotoxins and its producer, their applications in the practice of foods and feeds have been restricted due to insufficient evidence for safety and probiotic characteristics. In this study, an antibiotic susceptibility test was also conducted. Results showed that the strain LPB‐18 was susceptible to gentamicin, tetracycline, and penicillin and very susceptible to ampicillin, vancomycin, and rifampicin antibiotics with inhibition zones ranging between 33 and 55 mm. Therefore, *B. amyloliquefaciens* LPB‐18 could apply to reducing the concentrations of mycotoxins in animal feedstuffs.

## 5 CONCLUSIONS

In this study, a new *Bacillus* strain with excellent antimicrobial properties and probiotic attributions named LPB‐18 was isolated from the breeding soil of earthworm and identified evolution footprint closer to the species of *B. amyloliquefaciens*. The antimicrobial properties of strain LPB‐18 show various antimicrobial activities against food‐borne pathogens with multidrug resistance, especially filamentous fungi. Subsequently, molecular verification of lipopeptides synthase gene, the RT‐HPLC and MALDI‐TOF MS comprehensively showed the purified antimicrobial compounds, which contained mono‐lipopeptides of fengycin isoforms, fengycin A (C_16_–C_17_) and fengycin B (C_15_–C_17_). Besides, antibiotics susceptibility tests, tolerance of acidic conditions, and bile salts assay also demonstrated that strain LPB‐18 would be a potential probiotic. We report that the fengycin produced by *B. amyloliquefaciens* LPB‐18 is a promising prospect as a candidate for biological strain in agriculture commodities and animal feedstuffs.

## CONFLICT OF INTEREST

The authors declare that they have no conflict of interest.
